# Dual Competing Photovoltaic Supply Chains: A Social Welfare Maximization Perspective

**DOI:** 10.3390/ijerph14111416

**Published:** 2017-11-20

**Authors:** Zhisong Chen, Shong-Iee Ivan Su

**Affiliations:** 1 Business School, Nanjing Normal University, Qixia District, Nanjing 210023, China; 2 Supply Chain and Logistics Management Research Lab, Department of Business Administration, School of Business, Soochow University, Taipei 11102, Taiwan; sisu@scu.edu.tw

**Keywords:** dual PV supply chains, game-theoretical models, supply chain strategies, cooperation, subsidy, social welfare maximization

## Abstract

In the past decades, the inappropriate subsidy policies in many nations have caused problems such as serious oversupply, fierce competition and subpar social welfare in the photovoltaic (PV) industry in many nations. There is a clear shortage in the PV industry literature regarding how dual supply chains compete and the key decision issues regarding the competition between dual PV supply chains. It is critical to develop effective subsidy policies for the competing PV supply chains to achieve social welfare maximization. This study has explored the dual PV supply chain competition under the Bertrand competition assumption by three game-theoretical modeling scenarios (or supply chain strategies) considering either the public subsidy or no subsidy from a social welfare maximization perspective. A numerical analysis complemented by two sensitivity analyses provides a better understanding of the pricing and quantity decision dynamics in the dual supply chains under three different supply chain strategies and the corresponding outcomes regarding the total supply chain profits, the social welfare and the required total subsidies. The key findings disclose that if there are public subsidies, the dual PV supply chains have the strongest intention to pursue the decentralized strategy to achieve their maximal returns rather than the centralized strategy that would achieve the maximal social welfare; however, the government would need to pay for the maximal subsidy budget. Thus, the best option for the government would be to encourage the dual PV supply chains to adopt a centralized strategy since this will not only maximize the social welfare but also, at the same time, minimize the public subsidy. With a smart subsidy policy, the PV industry can make the best use of the subsidy budget and grow in a sustainable way to support the highly demanded solar power generation in many countries trying very hard to increase the proportion of their clean energy to combat the global warming effect. Several subsidy policies such as shared solar energy arrangements and performance-based incentive (PBI) are proposed to integrate the market users and the PV supply chains. This study serves as a pioneering study into the dual PV supply chain research which is very limited in the PV management and policy study literature. The findings and several untended issues provide a foundation for the future PV supply chain studies.

## 1. Introduction

Given the abundance of sunshine across the globe, solar power has the potential to supply a significant amount of electricity that is both economically and environmentally attractive. Solar power generates electricity with no global warming pollution, no fuel costs, and no risks of fuel price spikes, and has the potential to help move the world toward cleaner, reliable, and affordable sources of electricity [1,2]. The installed price of solar energy has declined significantly in recent years as policy and market forces have driven more and more solar installations.

The solar photovoltaic energy, a major source of solar power, has been encouraged and developed by many countries to counter the impact due to the global warming effect. Photovoltaics (PV) industry policies are made and implemented by many governments. Large-scale photovoltaic energy development programs can be seen in many developed and developing nations. Furthermore, costs for large-scale PV projects have dropped significantly since 2010 owning to the reductions in technology prices, innovative financing, and growing networks of solar installers and financial partners. By 2050, it is estimated the PV global cumulative installed capacity could reach 16% of the global electricity production [1]. On the one hand, this has led to the increase of the solar energy conversion efficiency and the reduction of the cost of PV modules (due to the advancement of solar technology and higher competition); on the other hand, it has also caused the oversupply and excess capacity of the PV module production.

According to the global market outlook for solar power [2], a total of 76.6 GW of solar was installed and connected to the grid in 2016, which was a 50% year-on-year growth over the 51.2 GW added in 2015. The global solar market in 2016 was even more dominated by China than it was the year before, China connected 34.5 GW to the grid, a 128% increase over the 15.1 GW it added the year before [2]. The United States was the world’s second largest solar power market in 2016, whose annual installed capacity was up 97% year-on-year, resulting in 14.8 GW, compared to 7.5 GW in 2015 [2]. At 8.6 GW newly added capacity, Japan was still the world’s third largest solar market in 2016 [2]. Solar in Europe continued its several-year long downward trend in 2016, adding 6.7 GW, a 21% decrease compared to the 8.6 GW installed in 2015 [2].

Obviously, China has become the world’s largest user market for PV system products. However, the consistent subsidy policy and the excessive profit incentive have caused the overcapacity and oversupply problems for the PV module productions leading to even more fierce competition. Furthermore, a large proportion of the PV modules/systems produced in China are exported to the foreign markets causing several anti-dumping investigations from the imported countries, mainly U.S. and European Union. Even with a continuous demand and rapid growth for the PV solar energy infrastructure developments, the oversupply issue continues creating a polarized competition among the PV supply chains.

PV industry is different from the other industries in the world, and the unique characteristics of the PV industry include: (i) the PV industry is promoting the transformation of human energy structure, reducing greenhouse gas emissions in the energy production and consumption, and benefiting sustainable development of mankind; (ii) the development of the PV industry can produce positive externalities, reduce carbon emissions and carbon tax, and produce environmental performance; (iii) the PV industry is a high-tech, high-investment and long-payback-period industry with a certain public welfare, whose development cannot be separated from the government subsidies and support, especially during its growing period; (iv) till now, the PV industry has become a highly competitive industry, no matter the production and supply of high-purity silicon in the upstream of PV industry, the manufacturing and supply of PV components and modules and the corresponding BoS products in the midstream, or the assembly, installation and operations of PV systems and its corresponding solutions of various PV power stations or plants in the downstream. In summary, due to the characteristics of sustainable development, environmental performance and public-welfare, the proper subsidy policy, the optimal operations mechanism and the competition strategies for the competing PV supply chains become important issues to be studied and solved.

Since the PV system applications have become a major source of the renewable and sustainable energy, its development facilitated by the public subsidy is well justified. How should the government subsidize competing PV supply chains to achieve the social welfare maximization rather than to cause the oversupply in the PV supply chains? Under the public subsidy policy, what would be the proper mechanism for the photovoltaic assembler (PA) and its C-Pi module supplier (MS) to follow when there are dual (i.e., two) PV supply chains competing with each other? These questions will be studied through a two-stage game/bargaining-theoretical modeling approach and the relevant numerical analyses under the decentralized, the centralized and the hybrid scenarios to compare the optimal decisions and performance outcomes with the social welfare maximization goal.

In the following sections, Section 2 will review relevant literatures. Section 3 will define a dual PV supply chain system assuming a Bertrand competition and the modeling assumptions in this study. Section 4.1 will develop a Stackelberg game model for the dual competing PV supply chains to analyze the decentralized decision-making scenario in the supply chains. Section 4.2 will formulate a Nash Bargaining model for the dual competing PV supply chains to analyze the centralized decision-making scenario in the supply chains. Section 4.3 will combine a Stackelberg game model and a Nash Bargaining model for the dual competing PV supply chains to analyze the hybrid decision-making scenario in the supply chains. Section 5 will develop and analyze the dual PV supply chain models without the subsidies, serving for the comparison study purpose to find out the impact of the subsidy policy. Section 6 uses a popular solar PV system and the world PV industry data to conduct the numerical and sensitivity analyses to assess the impact of the key supply chain parameters on the supply chain models developed in Section 4 and Section 5. The research findings and insights will also be summarized. Section 7 discusses the business and policy implications derived from our analysis. In the end, Section 8 concludes this study.

## 2. Literature Review

Our study is closely related to the following two research streams: the PV industry policies and related impacts, and the optimal operations management of PV supply chains.

Regarding the first stream, due to the growing research interest in the solar energy development, many recent literatures investigating PV industry policies and related impacts need to be mentioned. De Boeck et al. [3] carried out a comprehensive evaluation of the support policy for photovoltaic installations in the residential sector of the major European markets, and conducted a comparison of past and present policies in the major EU market through investment profitability. Huang [4] studied the effects of the China and U.S. subsidy policies through comparing their developing strategies, specific subsidies and industrial conditions using two three-stage sequential game models, identified the inherent defects of China’s subsidy and analyzed the advantages of the U.S. industrial and trade policy portfolio. Zhi et al. [5] examined and compared the history of the China PV industry policy development to those of the United States, Germany and Japan from the perspective of both the supply-side and the demand-side policies. Chen [6] studied China’s central-local government relations during the formation and implementation of the PV industry policies.

Furthermore, also on the second stream, the optimal operations management of PV supply chains. Only until very recent, the study of optimal operations management of PV supply chains have been paid attention to, and the literatures regarding the optimal operations management of PV supply chain studies at the firm or inter-firm level can be found and are growing very fast. Davies and Joglekar [7] studied the market value for the integration of a PV supply chain. Marsillac [8] builds a framework of the PV supply chain, compares critical components of the PV supply chain, and identifies the research gaps and common problems in the PV supply chain. Bergesen and Suh [9] proposes a mathematical framework to distinguish the effects of learning on the direct inputs to a technology from the effects of learning on value added, and incorporates those effects throughout the PV supply chain of a technology using a life cycle assessment framework. Using a multiple case study method, Besiou and Van Wassenhove [10] analyzed the closed-loop PV supply chains involving key stakeholders in the design, production, collection, and recovery of PV panels, and identified the critical factors affecting the reverse supply chain management of the used panels. Kim and Jeong [11] analyzes and compares three real cases of manufacturer’s recycling policy, from the perspective of a closed-loop supply chain, develops two mathematical models to help PV system manufacturers for the supply chain planning and choosing the suitable recycling policies under different circumstances. Chen and Su [12] explored a coordination mechanism for the PV supply chain considering the strategic consumers’ behavior under the government’s subsidy policies and considered its impact on the decisions of the supply chain stakeholders in China. After the analysis of several game theoretic decision models and their numerical studies, Chen and Su [13] developed a joint decision-making method for the core members in a single PV supply chain considering the government subsidy. Dehghani, Jabalameli and Jabbarzadeh [14] developed a two-phase approach based on DEA and the robust optimization models to design and plan a solar photovoltaic supply chain in an uncertain environment. Zhang and Wang [15] studied a price competition model of an environmentally friendly supply chain vs. a harmful competing supply chain consisting of one manufacturer and one retailer from the perspective of the corporation social responsibility (CSR), adopting a two-stage Stackelberg game under the condition of incomplete knowledge and validating the results through a case in the PV industry.

However, these recent PV supply chain studies considered only a single supply chain scenario. Since there is an obvious trend that in many industries it is no longer competition among firms rather it is competition among supply chains [16,17,18], there is a clear shortage in the PV industry literature regarding how dual supply chains compete and the key decision issues regarding the competition between dual PV supply chains. This paper intends to address the literature shortage issue and hopes to shed some lights on the competition of dual supply chains in the PV industry under the public subsidy.

Bertrand’s model of strategic interaction between competing firms has become the primary workhorse for the analysis of imperfect competition and appears in the studies of a variety of fields, notably industrial organization and international trade [19,20,21,22].

In the PV industry, many competing firms are still producing similar products with similar cost structures. This characteristics resembles to the properties of a Bertrand competition model. Thus, this paper will explore the dual supply chain competition and cooperation considering the social welfare maximization goal in the PV industry assuming Bertrand competition.

## 3. Dual Competing Photovoltaic Supply Chains and Modeling Assumptions

**Figure 1:**
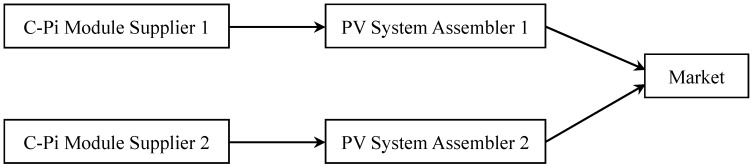
Dual competing PV supply chains.

The core component of a solar photovoltaic system (i.e., a PV system) is the solar module or panel. The manufacturing process of solar modules includes: C-Pi purification, ingot molding, wafer slicing, cells manufacturing and panel/module producing. The solar modules together with batteries, controllers, inverters and trackers are then assembled as a PV system ready for the commercial or residential installation. Therefore, a PV supply chain is mainly composed of a photovoltaic assembler (will be called assembler or PA in this paper) and its C-Pi module supplier (will be called supplier or MS). In practice, a large PA normally sources from a large MS who does not supply to the competing PAs of a similar market scale. In North American market, for example, Solar City, one of the major PAs, sources the modules/panels from a major Asian MS, while its key competitor, Canadian Solar, sources the modules/panels from another Asian MS. Figure 1 shows there are thus dual PV supply chains competing in a PV system market providing homogenous products and corresponding services. In a dual competing PV supply chains, two independent PV supply chains compete in the market with the differentiated PV system product via price-competition strategy.

Both PV supply chains may adopt a pure decentralized strategy, i.e., the members in a PV supply chain compete in a Stackelberg way [23,24,25,26], a pure centralized strategy, i.e., the members in a PV supply chain cooperate to make joint decisions, or a hybrid strategy where one PV supply chain adopting a decentralized strategy while the other adopts a centralized strategy. For convenience, the notation *i*, *j* = 1, 2 (*i* ≠ *j* ), is introduced to indicated variables and parameters. The cost of a module produced by supplier *i* is $c_{i}$. The Balance-of-System (BoS) cost of a PV system assembly for assembler *i* is $c_{0i}$, which includes the equipment cost of batteries, controllers, inverters and trackers, as well as expenses of civil engineering, installation engineering, project design and engineering acceptance expenses and prophase related expenses. The wholesale price of a module produced by supplier *i* is $w_{i}$. The retail price of a PV system in the market by assembler *i* is $p_{i}$. The demand of PV systems in the market for assembler *i* is $q_{i}$. The inverse demand functions of PV systems in the market is given by: $p_{i}\left(q_{i},q_{j}\right)=\alpha -\beta q_{i}-\gamma q_{j}$, where α is the price cap, *β* is the reaction extent of the retail price w.r.t. the change of demand, and *γ* ∈ [0, 1] measures the degree of product substitutability, with *γ* = 0 and 1 representing the cases of completely independent and homogenous products, respectively. Besides, β>γ. Letting a=α/(β+γ), $b=\beta /\left(\beta^{2}-\gamma^{2}\right)$, $d=\gamma /\left(\beta^{2}-\gamma^{2}\right)$, we can write the direct demand function of PV systems as $q_{i}\left(p_{i},p_{j}\right)=a-bp_{i}+dp_{j}$, where *a* is the maximum demand (when its own retail price and its competitor’s retail price are both zero), *b* is the reaction extent of the PV system demand w.r.t. the change of its own retail price, *d* is the reaction extent of the PV system demand w.r.t. the change of its competitor’s retail price, *b* > *d*. The decision variables are the wholesale price $w_{i}$ of a module and the retail price $p_{i}$ of a PV system. The government subsidy factor of per-unit standard PV system for the PV system assembler is s, and the total subsidy from the government is $TS=s\left(q_{i}+q_{j}\right)$. The decision variables are the wholesale price $w_{i}$ of a module, the retail price $p_{i}$ of a PV system, and the government’s subsidy factor s.

The profit functions of the PV supply chain *SC_i_* and *SC_j_*, the profit functions of the PV system assembler *PA_i_* and *PA_j_*, and the profit functions of the module supplier *MS_i_* and *MS_j_* are represented as follows:

(1) $$\Pi_{SC_{i}}\left(p_{i}\right)=\left(p_{i}-c_{0i}-c_{i}+s\right)\left(a-bp_{i}+dp_{j}\right)$$

(2) $$\Pi_{PA_{i}}\left(p_{i}\right)=\left(p_{i}-c_{0i}-w_{i}+s\right)\left(a-bp_{i}+dp_{j}\right)$$

(3) $$\Pi_{MS_{i}}\left(w_{i}\right)=\left(w_{i}-c_{i}\right)\left(a-bp_{i}+dp_{j}\right)$$

(4) $$\Pi_{SC_{j}}\left(p_{j}\right)=\left(p_{j}-c_{0j}-c_{j}+s\right)\left(a-bp_{j}+dp_{i}\right)$$

(5) $$\Pi_{PA_{j}}\left(p_{j}\right)=\left(p_{j}-c_{0j}-w_{j}+s\right)\left(a-bp_{j}+dp_{i}\right)$$

(6) $$\Pi_{MS_{j}}\left(w_{j}\right)=\left(w_{j}-c_{j}\right)\left(a-bp_{j}+dp_{i}\right)$$

Following Singh and Vives (1984), the representative consumer surplus *CS* for the dual competing PV supply chains can be defined as follows [27]:

(7) $$CS\left(q_{i},q_{j}\right)=\frac{a}{b-d}\left(q_{i}+q_{j}\right)-\frac{b}{2\left(b^{2}-d^{2}\right)}{\left(q_{i}+q_{j}\right)}^{2}+\frac{1}{b+d}q_{i}q_{j}-\left(p_{i}q_{i}+p_{j}q_{j}\right)$$

According to the classical economics theory, social welfare is the sum of the consumer surplus and the producer surplus in the market. In our study, social welfare for the dual competing PV supply chains is the sum of the consumer surplus and the dual PV supply chains surplus minus the government subsidies in the market. Hence, we can express the social welfare function SW for the dual competing PV supply chains as follows [27]:

(8) $$SW\left(q_{i},q_{j}\right)=\frac{a}{b-d}\left(q_{i}+q_{j}\right)-\frac{b}{2\left(b^{2}-d^{2}\right)}{\left(q_{i}+q_{j}\right)}^{2}+\frac{1}{b+d}q_{i}q_{j}-\left[\left(c_{i}+c_{0i}\right)q_{i}+\left(c_{j}+c_{0j}\right)q_{j}\right]$$

Game theoretic models with Nash bargaining mechanism [28,29,30,31] are developed to study the equilibrium solutions, i.e., constant solutions to differential equations of systems to ensure that all competing influences are balanced regarding the PV supply chain cooperation under dual PV supply chain competition and the social welfare maximization.

To study the dual competing PV supply chain problem under the social welfare maximization goal, the game theoretic models with Nash bargaining mechanism [28,29,30,31] are developed to obtain the equilibrium solutions regarding the dual PV supply chain cooperation, i.e., constant solutions to the differential equations of systems to ensure that all competing influences are balanced regarding the PV supply chain cooperation under dual PV supply chain competition and the social welfare maximization (Section 4 and Section 5). It is assumed that the dual PV supply chains compete in a Bertrand way [19,20,21,22], i.e., the dual supply chains compete on the retail price of the PV system they sell and they make pricing decisions independently but at the same time.

In a decentralized PV supply chain, the government decides the optimal subsidy factor *s* to achieve the social welfare maximization, a module supplier (MS) and a PV system assembler (PA) make optimal decisions in a Stackelberg way [23,24,25,26], that is, module supplier *i* chooses his optimal wholesale price $w_{i}$ first, then PV system assembler *i* chooses his optimal retail price $p_{i}$ under the Bertrand competition in the dual PV supply chains. We note that there is no cooperation between a supplier and an assembler in a decentralized supply chain. In contrast, in a centralized PV supply chain, even though the government still decides the optimal subsidy factor *s* to achieve the social welfare maximization, module supplier *i* now cooperates with PV assembler *i* to make joint decisions; they will bargain over the wholesale price $w_{i}$, then determine the optimal retail price $p_{i}$ under the Bertrand competition in the dual PV supply chains. Three supply chain modeling scenarios will be investigated for this study: (1) a *decentralized* scenario: both competing supply chains adopt a decentralized strategy; (2) a *centralized* scenario: both competing supply chains adopt a centralized strategy; and (3) a *hybrid* scenario: one supply chain adopts a centralized strategy while the other supply chain adopts a decentralized strategy.

## 4. Dual Competing PV Supply Chain Equilibrium and Cooperation under the Social Welfare Maximization

The game-theoretical decision models regarding the module suppliers (MSs) and the PV system assemblers (PAs) in the dual competing PV supply chains are developed in this section. The optimal conditions for three modeling scenarios are derived through the theoretical modeling analyses resulting in the equilibrium (or optimal) wholesale price and retail price for each scenario. Thus, the profits of the dual PV supply chains, MSs, PAs and the social welfare can be calculated for the numerical analysis study in the next section and also compared.

For the convenience of notation, let x=b+d, $\bar{x}=b-d$, $X=x\bar{x}=b^{2}-d^{2}$; y=2b+d, $\bar{y}=2b-d$, $Y=y\bar{y}=4b^{2}-d^{2}$; $Z=2b^{2}-d^{2}$; V=bd; $W=b^{2}$; $M=\left(b\bar{x}\right)/\left(2\bar{y}\right)$, $N=\left[b\bar{x}\left(2Z+V\right)\right]/\left(2\bar{y}Z\right)$. In the following sections, these notations will be used to mark parameters in the analytical models.

### 4.1. Decentralized Modeling Scenario: Dual Competing PV Supply Chain Equilibrium under the Social Welfare Maximization

In the decentralized modeling scenario, the government announces the subsidy factor first, then, the PV system assembler *PA_i_* and the module supplier *MS_i_* play the Stackelberg game, so do *MS_j_* and *PA_j_*. *MS_i_* acts as a Stackelberg leader and *PA_i_* acts as a Stackelberg follower, so do *MS_j_* and *PA_j_*. *MS_i_* first decides the wholesale price of a module, *PA_i_* then decides the retail price of a PV system to maximize his profit, so do *MS_j_* and *PA_j_*. Finally, the retail price competition occurs in the PV market. The Stackelberg game model for the dual competing PV supply chains can be formulated as:

(9) $$\begin{array}{c} \max_{s}\;SW\left(q_{i}\left(w_{i}^{d}\left(s\right),w_{j}^{d}\left(s\right),s\right),q_{j}\left(w_{j}^{d}\left(s\right),w_{i}^{d}\left(s\right),s\right)\right)\\ w_{i}^{d}\left(s\right)\;and\;w_{j}^{d}\left(s\right)\;are\;derived\;from\;solving\;the\;following\;problem\\ s.t.\left\{ \begin{array}{c} \left\{ \begin{array}{c} \max_{w_{i}}\;\Pi_{MS_{i}}\left(w_{i},q_{i}\left(p_{i}^{d}\left(w_{i},w_{j}\right),p_{j}^{d}\left(w_{j},w_{i}\right)\right)\right)\\ \max_{w_{j}}\;\Pi_{MS_{j}}\left(w_{j},q_{j}\left(p_{j}^{d}\left(w_{j},w_{i}\right),p_{i}^{d}\left(w_{i},w_{j}\right)\right)\right) \end{array}\right.\\ p_{i}^{d}\left(w_{i},w_{j}\right)\;and\;p_{j}^{d}\left(w_{j},w_{i}\right)\;are\;derived\;from\;solving\;the\;following\;problem\\ s.t.\left\{ \begin{array}{c} \max_{p_{i}}\;\Pi_{PA_{i}}\left(p_{i}\right)\\ \max_{p_{j}}\;\Pi_{PA_{j}}\left(p_{j}\right) \end{array}\right. \end{array}\right. \end{array}$$

Solving this two-stage Stackelberg game, we can obtain the equilibrium government subsidy factor $s_{d}$, the equilibrium wholesale price $w_{i}^{d}$ and $w_{j}^{d}$, the equilibrium retail price $p_{i}^{d}$ and $p_{j}^{d}$, and the equilibrium order quantity $q_{i}^{d}$ and $q_{j}^{d}$ as follows:

(10) $$s_{d}=\frac{Z+W}{bZ}\left\{2a-\bar{x}\left[\left(c_{i}+c_{0i}\right)+\left(c_{j}+c_{0j}\right)\right]\right\}$$

(11) $$w_{i}^{d}=\frac{y}{2Z-V}\left(a+\bar{x}s_{d}\right)+\frac{Z}{4Z^{2}-V^{2}}\left[2Z\left(c_{i}+c_{0i}\right)+V\left(c_{j}+c_{0j}\right)\right]-c_{0i}$$

(12) $$w_{j}^{d}=\frac{y}{2Z-V}\left(a+\bar{x}s_{d}\right)+\frac{Z}{4Z^{2}-V^{2}}\left[2Z\left(c_{j}+c_{0j}\right)+V\left(c_{i}+c_{0i}\right)\right]-c_{0j}$$

(13) $$p_{i}^{d}=\frac{\left(Y+Z\right)a-Zbs_{d}}{\bar{y}\left(2Z-V\right)}+\frac{bZ\left[b\left(Y+2Z\right)\left(c_{i}+c_{0i}\right)+2d\left(Z+W\right)\left(c_{j}+c_{0j}\right)\right]}{Y\left(4Z^{2}-V^{2}\right)}$$

(14) $$p_{j}^{d}=\frac{\left(Y+Z\right)a-Zbs_{d}}{\bar{y}\left(2Z-V\right)}+\frac{bZ\left[b\left(Y+2Z\right)\left(c_{j}+c_{0j}\right)+2d\left(Z+W\right)\left(c_{i}+c_{0i}\right)\right]}{Y\left(4Z^{2}-V^{2}\right)}$$

(15) $$q_{i}^{d}=\frac{bZ}{\bar{y}\left(2Z-V\right)}\left(a+\bar{x}s_{d}\right)-\frac{bZ\left[\left(2Z^{2}-V^{2}\right)\left(c_{i}+c_{0i}\right)-VZ\left(c_{j}+c_{0j}\right)\right]}{Y\left(4Z^{2}-V^{2}\right)}$$

(16) $$q_{j}^{d}=\frac{bZ}{\bar{y}\left(2Z-V\right)}\left(a+\bar{x}s_{d}\right)-\frac{bZ\left[\left(2Z^{2}-V^{2}\right)\left(c_{j}+c_{0j}\right)-VZ\left(c_{i}+c_{0i}\right)\right]}{Y\left(4Z^{2}-V^{2}\right)}$$

We can get also the equilibrium profits of *PA_i_*, *MS_i_*, *SC_i_*, *PA_j_*, *MS_j_*, *SC_j_*, the equilibrium *SW*, *CS* and *TS* as follows:

(17) $$\Pi_{PA_{i}}^{d}=\frac{1}{b}{\left(q_{i}^{d}\right)}^{2}$$

(18) $$\Pi_{MS_{i}}^{d}=\frac{Y}{bZ}{\left(q_{i}^{d}\right)}^{2}$$

(19) $$\Pi_{SC_{i}}^{d}=\frac{Y+Z}{bZ}{\left(q_{i}^{d}\right)}^{2}$$

(20) $$\Pi_{PA_{j}}^{d}=\frac{1}{b}{\left(q_{j}^{d}\right)}^{2}$$

(21) $$\Pi_{MS_{j}}^{d}=\frac{Y}{bZ}{\left(q_{j}^{d}\right)}^{2}$$

(22) $$\Pi_{SC_{j}}^{d}=\frac{Y+Z}{bZ}{\left(q_{j}^{d}\right)}^{2}$$

(23) $$SW_{d}=\frac{a}{\bar{x}}\left(q_{i}^{d}+q_{j}^{d}\right)-\frac{b}{2X}{\left(q_{i}^{d}+q_{j}^{d}\right)}^{2}+\frac{1}{x}q_{i}^{d}q_{j}^{d}-\left[\left(c_{i}+c_{0i}\right)q_{i}^{d}+\left(c_{j}+c_{0j}\right)q_{j}^{d}\right]$$

(24) $$CS_{d}=\frac{a}{\bar{x}}\left(q_{i}^{d}+q_{j}^{d}\right)-\frac{b}{2X}{\left(q_{i}^{d}+q_{j}^{d}\right)}^{2}+\frac{1}{x}q_{i}^{d}q_{j}^{d}-\left(p_{i}^{d}q_{i}^{d}+p_{j}^{d}q_{j}^{d}\right)$$

(25) $$TS_{d}=s_{d}\left(q_{i}^{d}+q_{j}^{d}\right)$$

### 4.2. Centralized Modeling Scenario: Dual Competing PV Supply Chain Cooperation under the Social Welfare Maximization

In the centralized modeling scenario, the government announces the subsidy factor first, then, the module supplier *MS_i_* and the PV system assembler *PA_i_* cooperate with each other via Nash bargaining mechanism, so do *MS_j_* and *PA_j_*. *MS_i_* and *PA_i_* bargain over the wholesale price of a module to achieve cooperation within the PV supply chain *i*. The PV supply chain *i* then sets the retail price of a PV system to maximize its profit, so do *MS_j_* and *PA_j_*. At last, the retail price competition occurs in the PV market. The Stackelberg game-Nash bargaining model for the dual competing PV supply chains can be formulated as:

(26) $$\begin{array}{c} \max_{s}\;SW\left(q_{i}\left(p_{i}^{c}\left(s\right),p_{j}^{c}\left(s\right),s\right),q_{j}\left(p_{j}^{c}\left(s\right),p_{i}^{c}\left(s\right),s\right)\right)\\ s.t.\left\{ \begin{array}{c} w_{i}^{c}\left(s\right)\;and\;w_{j}^{c}\left(s\right)\;are\;derived\;from\;solving\;the\;following\;problem\\ \left\{ \begin{array}{c} \max_{w_{i}}\;{\left[\Pi_{MS_{i}}^{c}\left(w_{i}\right)\right]}^{\tau }{\left[\Pi_{PA_{i}}^{c}\left(w_{i}\right)\right]}^{1-\tau }\\ s.t.\Pi_{MS_{i}}^{c}\left(w_{i}\right)+\Pi_{PA_{i}}^{c}\left(w_{i}\right)=\Pi_{SC_{i}}^{c}\left(s\right)\\ \max_{w_{j}}\;{\left[\Pi_{MS_{j}}^{c}\left(w_{j}\right)\right]}^{\tau }{\left[\Pi_{PA_{j}}^{c}\left(w_{j}\right)\right]}^{1-\tau }\\ s.t.\Pi_{MS_{j}}^{c}\left(w_{j}\right)+\Pi_{PA_{j}}^{c}\left(w_{j}\right)=\Pi_{SC_{j}}^{c}\left(s\right) \end{array}\right.\\ p_{i}^{c}\left(s\right),p_{j}^{c}\left(s\right),\Pi_{SC_{i}}^{c}\left(s\right)\;and\;\Pi_{SC_{j}}^{c}\left(s\right)\;are\;derived\;from\;solving\;the\;following\;problem\\ \left\{ \begin{array}{c} \max_{p_{i}}\;\Pi_{SC_{i}}\left(p_{i}\right)\\ \max_{p_{j}}\;\Pi_{SC_{j}}\left(p_{j}\right) \end{array}\right. \end{array}\right. \end{array}$$

Solving this two-stage Stackelberg game-Nash bargaining problem, we can obtain the equilibrium government subsidy factor $s_{c}$, the bargaining wholesale price $w_{i}^{c}$ and $w_{j}^{c}$, the optimal retail price $p_{i}^{c}$ and $p_{j}^{c}$, and the optimal order quantity $q_{i}^{c}$ and $q_{j}^{c}$ as follows:

(27) $$s_{c}=\frac{a}{b}-\frac{\bar{x}}{2b}\left[\left(c_{i}+c_{0i}\right)+\left(c_{j}+c_{0j}\right)\right]$$

(28) $$w_{i}^{c}=\frac{\tau }{b}\left\{a-\frac{1}{2y}\left[\left(Z+W\right)\left(c_{i}+c_{0i}\right)+\left(X-2V\right)\left(c_{j}+c_{0j}\right)\right]\right\}+c_{i}$$

(29) $$w_{j}^{c}=\frac{\tau }{b}\left\{a-\frac{1}{2y}\left[\left(Z+W\right)\left(c_{j}+c_{0j}\right)+\left(X-2V\right)\left(c_{i}+c_{0i}\right)\right]\right\}+c_{j}$$

(30) $$p_{i}^{c}=\frac{1}{2y}\left[\left(b+y\right)\left(c_{i}+c_{0i}\right)+x\left(c_{j}+c_{0j}\right)\right]$$

(31) $$p_{j}^{c}=\frac{1}{2y}\left[\left(b+y\right)\left(c_{j}+c_{0j}\right)+x\left(c_{i}+c_{0i}\right)\right]$$

(32) $$q_{i}^{c}=a-\frac{\left(Z+W\right)\left(c_{i}+c_{0i}\right)+\left(X-2V\right)\left(c_{j}+c_{0j}\right)}{2y}$$

(33) $$q_{j}^{c}=a-\frac{\left(Z+W\right)\left(c_{j}+c_{0j}\right)+\left(X-2V\right)\left(c_{i}+c_{0i}\right)}{2y}$$

We can also get the optimal (or bargaining) profits of *PA_i_*, *MS_i_*, *SC_i_*, *PA_j_*, *MS_j_*, *SC_j_*, the equilibrium *SW*, *CS* and *TS* as follows:

(34) $$\Pi_{PA_{i}}^{c}=\frac{1-\tau }{b}{\left(q_{i}^{c}\right)}^{2}$$

(35) $$\Pi_{MS_{i}}^{c}=\frac{\tau }{b}{\left(q_{i}^{c}\right)}^{2}$$

(36) $$\Pi_{SC_{i}}^{c}=\frac{1}{b}{\left(q_{i}^{c}\right)}^{2}$$

(37) $$\Pi_{PA_{j}}^{c}=\frac{1-\tau }{b}{\left(q_{j}^{c}\right)}^{2}$$

(38) $$\Pi_{MS_{j}}^{c}=\frac{\tau }{b}{\left(q_{j}^{c}\right)}^{2}$$

(39) $$\Pi_{SC_{j}}^{c}=\frac{1}{b}{\left(q_{j}^{c}\right)}^{2}$$

(40) $$SW_{c}=\frac{a}{\bar{x}}\left(q_{i}^{c}+q_{j}^{c}\right)-\frac{b}{2X}{\left(q_{i}^{c}+q_{j}^{c}\right)}^{2}+\frac{1}{x}q_{i}^{c}q_{j}^{c}-\left[\left(c_{i}+c_{0i}\right)q_{i}^{c}+\left(c_{j}+c_{0j}\right)q_{j}^{c}\right]$$

(41) $$CS_{c}=\frac{a}{\bar{x}}\left(q_{i}^{c}+q_{j}^{c}\right)-\frac{b}{2X}{\left(q_{i}^{c}+q_{j}^{c}\right)}^{2}+\frac{1}{x}q_{i}^{c}q_{j}^{c}-\left(p_{i}^{c}q_{i}^{c}+p_{j}^{c}q_{j}^{c}\right)$$

(42) $$TS_{c}=s_{c}\left(q_{i}^{c}+q_{j}^{c}\right)$$

### 4.3. Hybrid Modeling Scenario: Dual Competing PV Supply Chain Equilibrium and Cooperation under the Social Welfare Maximization

In the hybrid modeling scenario, the government announces the subsidy factor first, then, the PV system assembler *PA_i_* and the module supplier *MS_i_* play a Stackelberg game, *MS_i_* acts as a Stackelberg leader and *PA_i_* acts as a Stackelberg follower; *PA_j_* and *MS_j_* cooperate with each other via a Nash bargaining mechanism. *MS_i_* first decides the wholesale price of a module, *PA_i_* then decides the retail price of a PV system to maximize his profit; *MS_j_* and *PA_j_* bargain over the wholesale price of a module to achieve cooperation within the PV supply chain *j*. The PV supply chain *j* then decides the retail price of a PV system to maximize its profit. At last, the retail price competition occurs in the PV market. The Stackelberg game-Nash bargaining model for the dual competing PV supply chains can be formulated as:

(43) $$\begin{array}{c} \max_{s}\;SW\left(q_{i}\left(p_{i}^{d}\left(s\right),p_{j}^{c}\left(s\right),s\right),q_{j}\left(p_{j}^{c}\left(s\right),p_{i}^{d}\left(s\right),s\right)\right)\\ s.t.\left\{ \begin{array}{c} w_{j}^{c}\left(s\right)\;is\;derived\;from\;solving\;the\;following\;problem\\ \left\{ \begin{array}{c} \max_{w_{j}}\;{\left[\Pi_{MS_{j}}^{c}\left(w_{j}\right)\right]}^{\tau }{\left[\Pi_{PA_{j}}^{c}\left(w_{j}\right)\right]}^{1-\tau }\\ s.t.\Pi_{MS_{j}}^{c}\left(w_{j}\right)+\Pi_{PA_{j}}^{c}\left(w_{j}\right)=\Pi_{SC_{j}}^{c}\left(s\right) \end{array}\right.\\ p_{i}^{d}\left(s\right),p_{j}^{c}\left(s\right),w_{i}^{d}\left(s\right)and\;\Pi_{SC_{j}}^{c}\left(s\right)\;are\;derived\;from\;solving\;the\;following\;problem\\ \left\{ \begin{array}{c} \max_{w_{i}}\;\Pi_{MS_{i}}\left(w_{i}\right)\\ s.t.\left\{ \begin{array}{c} \underset{p_{i}}{\max}\;\Pi_{PA_{i}}\left(p_{i}\right)\\ \underset{p_{j}}{\max}\;\Pi_{SC_{j}}\left(p_{j}\right) \end{array}\right. \end{array}\right. \end{array}\right. \end{array}$$

Solving this two-stage Stackelberg game-Nash bargaining problem, we can obtain the equilibrium government subsidy factor $s_{cd}$, the equilibrium (or bargaining) wholesale price $w_{i}^{d}$ and $w_{j}^{c}$, the equilibrium (or optimal) retail price $p_{i}^{d}$ and $p_{j}^{c}$, and the equilibrium (or optimal) order quantity $q_{i}^{d}$ and $q_{j}^{c}$ as follows:

(44) $$s_{cd}=\frac{x\left\{M\left[a-\bar{x}\left(c_{i}+c_{0i}\right)\right]+N\left[a-\bar{x}\left(c_{j}+c_{0j}\right)\right]\right\}-\left[b\left(MA+NB\right)+d\left(MB+NA\right)\right]}{b{\left(M+N\right)}^{2}-2\bar{x}MN}$$

Hereinto,

$$A=\frac{b}{2Y}\left[ya-Z\left(c_{i}+c_{0i}\right)+V\left(c_{j}+c_{0j}\right)\right],$$

$$B=\frac{b}{2YZ}\left[y\left(2Z+V\right)a-\left(2Z^{2}-V^{2}\right)\left(c_{j}+c_{0j}\right)+ZV\left(c_{0i}+c_{i}\right)\right].$$

(45) $$w_{i}^{d}=\frac{1}{2Z}\left[y\left(a+\bar{x}s_{cd}\right)-Z\left(c_{0i}+c_{i}\right)+V\left(c_{0j}+c_{j}\right)\right]+c_{i}$$

(46) $$w_{j}^{c}=\frac{\tau }{2ZY}\left[y\left(2Z+V\right)\left(a+\bar{x}s_{cd}\right)-\left(2Z^{2}-V^{2}\right)\left(c_{j}+c_{0j}\right)+ZV\left(c_{0i}+c_{i}\right)\right]+c_{j}$$

(47) $$p_{i}^{d}=\frac{1}{YZ}\left\{y\left[\left(W+Z\right)a-\left(X+V\right)bs_{cd}\right]+WZ\left(c_{0i}+c_{i}\right)+\left(W+Z\right)V\left(c_{0j}+c_{j}\right)\right\}$$

(48) $$p_{j}^{c}=\frac{1}{2ZY}\left\{y\left[\left(2Z+V\right)a-\left(Y-V\right)bs_{cd}\right]+\left(2Z+Y\right)W\left(c_{0j}+c_{j}\right)+ZV\left(c_{0i}+c_{i}\right)\right\}$$

(49) $$q_{i}^{d}=\frac{b}{2Y}\left[y\left(a+\bar{x}s_{cd}\right)-Z\left(c_{0i}+c_{i}\right)+V\left(c_{0j}+c_{j}\right)\right]$$

(50) $$q_{j}^{c}=\frac{b}{2ZY}\left[y\left(2Z+V\right)\left(a+\bar{x}s_{cd}\right)-\left(2Z^{2}-V^{2}\right)\left(c_{0j}+c_{j}\right)+ZV\left(c_{0i}+c_{i}\right)\right]$$

We can also get the optimal (or bargaining) profits of *PA_i_*, *MS_i_*, *SC_i_*, *PA_j_*, *MS_j_*, *SC_j_*, the equilibrium *SW*, *CS* and *TS* as follows:

(51) $$\Pi_{PA_{i}}^{d}=\frac{1}{b}{\left[q_{i}^{d}\left(s_{cd}\right)\right]}^{2}$$

(52) $$\Pi_{MS_{i}}^{d}=\frac{Y}{bZ}{\left[q_{i}^{d}\left(s_{cd}\right)\right]}^{2}$$

(53) $$\Pi_{SC_{i}}^{d}=\frac{Y+Z}{bZ}{\left[q_{i}^{d}\left(s_{cd}\right)\right]}^{2}$$

(54) $$\Pi_{PA_{j}}^{c}=\frac{1-\tau }{b}{\left[q_{j}^{c}\left(s_{cd}\right)\right]}^{2}$$

(55) $$\Pi_{MS_{j}}^{c}=\frac{\tau }{b}{\left[q_{j}^{c}\left(s_{cd}\right)\right]}^{2}$$

(56) $$\Pi_{SC_{j}}^{c}=\frac{1}{b}{\left[q_{j}^{c}\left(s_{cd}\right)\right]}^{2}$$

(57) $$\begin{array}{cc} SW_{cd} & =\frac{a}{\bar{x}}\left[q_{i}^{d}\left(s_{cd}\right)+q_{j}^{c}\left(s_{cd}\right)\right]-\frac{b}{2X}{\left[q_{i}^{d}\left(s_{cd}\right)+q_{j}^{c}\left(s_{cd}\right)\right]}^{2}\\ & +\frac{1}{x}q_{i}^{d}\left(s_{cd}\right)q_{j}^{c}\left(s_{cd}\right)-\left[\left(c_{i}+c_{0i}\right)q_{i}^{d}\left(s_{cd}\right)+\left(c_{j}+c_{0j}\right)q_{j}^{c}\left(s_{cd}\right)\right] \end{array}$$

(58) $$CS_{cd}=\frac{a}{\bar{x}}\left(q_{i}^{d}+q_{j}^{c}\right)-\frac{b}{2X}{\left(q_{i}^{d}+q_{j}^{c}\right)}^{2}+\frac{1}{x}q_{i}^{d}q_{j}^{c}-\left(p_{i}^{d}q_{i}^{d}+p_{j}^{c}q_{j}^{c}\right)$$

(59) $$TS_{cd}=s_{cd}\left(q_{i}^{d}+q_{j}^{c}\right)$$

## 5. Comparison Analysis: No Subsidy

To validate the impact and the necessity of the public subsidy, the equilibrium and cooperation of the dual PV supply chains without the government’s subsidy, i.e., s=0, will be analyzed in this section.

### 5.1. Decentralized Modeling Scenario: Dual Competing PV Supply Chain Equilibrium without the Government’s Subsidy

In the decentralized modeling scenario, the PV system assembler *PA_i_* and the module supplier *MS_i_* play the Stackelberg game, so do *MS_j_* and *PA_j_*. *MS_i_* acts as a Stackelberg leader and *PA_i_* acts as a Stackelberg follower. *MS_i_* first decides the wholesale price of a module, *PA_i_* then decides the retail price of a PV system to maximize his profit, so do *MS_j_* and *PA_j_*. Finally, the retail price competition occurs in the PV market. The Stackelberg game model for the dual competing PV supply chains can be formulated as:

(60) $$\left\{ \begin{array}{c} \left\{ \begin{array}{c} \underset{w_{i}}{\max}\;\Pi_{MS_{i}}\left(w_{i},q_{i}\left(p_{i}^{d}\left(w_{i},w_{j}\right),p_{j}^{d}\left(w_{j},w_{i}\right)\right)\right)\\ \underset{w_{j}}{\max}\;\Pi_{MS_{j}}\left(w_{j},q_{j}\left(p_{j}^{d}\left(w_{j},w_{i}\right),p_{i}^{d}\left(w_{i},w_{j}\right)\right)\right) \end{array}\right.\\ p_{i}^{d}\left(w_{i},w_{j}\right),p_{j}^{d}\left(w_{j},w_{i}\right)\;are\;derived\;from\;solving\;the\;following\;problem\\ s.t.\left\{ \begin{array}{c} \underset{p_{i}}{\max}\;\Pi_{PA_{i}}\left(p_{i}\right)\\ \underset{p_{j}}{\max}\;\Pi_{PA_{j}}\left(p_{j}\right) \end{array}\right. \end{array}\right.$$

Solving this two-stage Stackelberg game, we can obtain the equilibrium wholesale price $w_{i}^{d\prime }$ and $w_{j}^{d\prime }$, the equilibrium retail price $p_{i}^{d\prime }$ and $p_{j}^{d\prime }$, and the equilibrium order quantity $q_{i}^{d\prime }$ and $q_{j}^{d\prime }$ as follows:

(61) $$w_{i}^{d\prime }=\frac{ya}{2Z-V}+\frac{Z}{4Z^{2}-V^{2}}\left[2Z\left(c_{i}+c_{0i}\right)+V\left(c_{j}+c_{0j}\right)\right]-c_{0i}$$

(62) $$w_{j}^{d\prime }=\frac{ya}{2Z-V}+\frac{Z}{4Z^{2}-V^{2}}\left[2Z\left(c_{j}+c_{0j}\right)+V\left(c_{i}+c_{0i}\right)\right]-c_{0j}$$

(63) $$p_{i}^{d\prime }=\frac{\left(Y+Z\right)a}{\bar{y}\left(2Z-V\right)}+\frac{bZ\left[b\left(Y+2Z\right)\left(c_{i}+c_{0i}\right)+2d\left(Z+W\right)\left(c_{j}+c_{0j}\right)\right]}{Y\left(4Z^{2}-V^{2}\right)}$$

(64) $$p_{j}^{d\prime }=\frac{\left(Y+Z\right)a}{\bar{y}\left(2Z-V\right)}+\frac{bZ\left[b\left(Y+2Z\right)\left(c_{j}+c_{0j}\right)+2d\left(Z+W\right)\left(c_{i}+c_{0i}\right)\right]}{Y\left(4Z^{2}-V^{2}\right)}$$

(65) $$q_{i}^{d\prime }=\frac{bZa}{\bar{y}\left(2Z-V\right)}-\frac{bZ\left[\left(2Z^{2}-V^{2}\right)\left(c_{i}+c_{0i}\right)-VZ\left(c_{j}+c_{0j}\right)\right]}{Y\left(4Z^{2}-V^{2}\right)}$$

(66) $$q_{j}^{d\prime }=\frac{bZa}{\bar{y}\left(2Z-V\right)}-\frac{bZ\left[\left(2Z^{2}-V^{2}\right)\left(c_{j}+c_{0j}\right)-VZ\left(c_{i}+c_{0i}\right)\right]}{Y\left(4Z^{2}-V^{2}\right)}$$

We can also get the equilibrium profits of *PA_i_*, *MS_i_*, *SC_i_*, *PA_j_*, *MS_j_*, *SC_j_*, *SW* and *CS* as follows:

(67) $$\Pi_{PA_{i}}^{d\prime }=\frac{1}{b}{\left(q_{i}^{d\prime }\right)}^{2}$$

(68) $$\Pi_{MS_{i}}^{d\prime }=\frac{Y}{bZ}{\left(q_{i}^{d\prime }\right)}^{2}$$

(69) $$\Pi_{SC_{i}}^{d\prime }=\frac{Y+Z}{bZ}{\left(q_{i}^{d\prime }\right)}^{2}$$

(70) $$\Pi_{PA_{j}}^{d\prime }=\frac{1}{b}{\left(q_{j}^{d\prime }\right)}^{2}$$

(71) $$\Pi_{MS_{j}}^{d\prime }=\frac{Y}{bZ}{\left(q_{j}^{d\prime }\right)}^{2}$$

(72) $$\Pi_{SC_{j}}^{d\prime }=\frac{Y+Z}{bZ}{\left(q_{j}^{d\prime }\right)}^{2}$$

(73) $$SW_{d}^{\prime }=\frac{a}{\bar{x}}\left(q_{i}^{d\prime }+q_{j}^{d\prime }\right)-\frac{b}{2X}{\left(q_{i}^{d\prime }+q_{j}^{d\prime }\right)}^{2}+\frac{1}{x}q_{i}^{d\prime }q_{j}^{d\prime }-\left[\left(c_{i}+c_{0i}\right)q_{i}^{d\prime }+\left(c_{j}+c_{0j}\right)q_{j}^{d\prime }\right]$$

(74) $$CS_{d}^{\prime }=\frac{a}{\bar{x}}\left(q_{i}^{d\prime }+q_{j}^{d\prime }\right)-\frac{b}{2X}{\left(q_{i}^{d\prime }+q_{j}^{d\prime }\right)}^{2}+\frac{1}{x}q_{i}^{d\prime }q_{j}^{d\prime }-\left(p_{i}^{d\prime }q_{i}^{d\prime }+p_{j}^{d\prime }q_{j}^{d\prime }\right)$$

### 5.2. Centralized Modeling Scenario: Dual Competing PV Supply Chain Cooperation without the Government’s Subsidy 

In the centralized modeling scenario, the module supplier *MS_i_* and the PV system assembler *PA_i_* cooperate with each other via Nash bargaining mechanism, so do *MS_j_* and *PA_j_*. *MS_i_* and *PA_i_* bargain over the wholesale price of a module to achieve cooperation within the PV supply chain *i*. The PV supply chain *i* then decides the retail price of a PV system to maximize its profit, so do *MS_j_* and *PA_j_*. At last, the retail price competition occurs in the PV market. The Stackelberg game-Nash bargaining model for the dual competing PV supply chains can be formulated as:

(75) $$\begin{array}{c} \left\{ \begin{array}{c} \underset{w_{i}}{\max}\;{\left[\Pi_{MS_{i}}^{c}\left(w_{i}\right)\right]}^{\tau }{\left[\Pi_{PA_{i}}^{c}\left(w_{i}\right)\right]}^{1-\tau }\\ s.t.\;\Pi_{MS_{i}}^{c}\left(w_{i}\right)+\Pi_{PA_{i}}^{c}\left(w_{i}\right)=\Pi_{SC_{i}}^{c}\left(s\right)\\ \underset{w_{j}}{\max}\;{\left[\Pi_{MS_{j}}^{c}\left(w_{j}\right)\right]}^{\tau }{\left[\Pi_{PA_{j}}^{c}\left(w_{j}\right)\right]}^{1-\tau }\\ s.t.\;\Pi_{MS_{j}}^{c}\left(w_{j}\right)+\Pi_{PA_{j}}^{c}\left(w_{j}\right)=\Pi_{SC_{j}}^{c}\left(s\right) \end{array}\right.\\ p_{i}^{c}\left(s\right),p_{j}^{c}\left(s\right),\Pi_{SC_{i}}^{c}\left(s\right)\;and\;\Pi_{SC_{j}}^{c}\left(s\right)\;are\;derived\;from\;solving\;the\;following\;problem\\ \left\{ \begin{array}{c} \underset{p_{i}}{\max}\;\Pi_{SC_{i}}\left(p_{i}\right)\\ \underset{p_{j}}{\max}\;\Pi_{SC_{j}}\left(p_{j}\right) \end{array}\right. \end{array}$$

Solving this two-stage Stackelberg game-Nash bargaining problem, we can obtain the bargaining wholesale price $w_{i}^{c\prime }$ and $w_{j}^{c\prime }$, the optimal retail price $p_{i}^{c\prime }$ and $p_{j}^{c\prime }$, and the optimal order quantity $q_{i}^{c\prime }$ and $q_{j}^{c\prime }$ as follows:

(76) $$w_{i}^{c\prime }=\frac{\tau }{\bar{y}}\left\{a-\frac{1}{y}\left[Z\left(c_{i}+c_{0i}\right)-V\left(c_{j}+c_{0j}\right)\right]\right\}+c_{i}$$

(77) $$w_{j}^{c\prime }=\frac{\tau }{\bar{y}}\left\{a-\frac{1}{y}\left[Z\left(c_{j}+c_{0j}\right)-V\left(c_{i}+c_{0i}\right)\right]\right\}+c_{j}$$

(78) $$p_{i}^{c\prime }=\frac{1}{\bar{y}}a+\frac{b}{Y}\left[2b\left(c_{i}+c_{0i}\right)+d\left(c_{j}+c_{0j}\right)\right]$$

(79) $$p_{j}^{c\prime }=\frac{1}{\bar{y}}a+\frac{b}{Y}\left[2b\left(c_{j}+c_{0j}\right)+d\left(c_{i}+c_{0i}\right)\right]$$

(80) $$q_{i}^{c\prime }=\frac{b}{\bar{y}}a-\frac{b}{Y}\left[Z\left(c_{i}+c_{0i}\right)-V\left(c_{j}+c_{0j}\right)\right]$$

(81) $$q_{j}^{c\prime }=\frac{b}{\bar{y}}a-\frac{b}{Y}\left[Z\left(c_{j}+c_{0j}\right)-V\left(c_{i}+c_{0i}\right)\right]$$

We can also get the optimal (or bargaining) profits of *PA_i_*, *MS_i_*, *SC_i_*, *PA_j_*, *MS_j_*, *SC_j_*, *SW* and *CS* as follows:

(82) $$\Pi_{PA_{i}}^{c\prime }=\frac{1-\tau }{b}{\left(q_{i}^{c\prime }\right)}^{2}$$

(83) $$\Pi_{MS_{i}}^{c\prime }=\frac{\tau }{b}{\left(q_{i}^{c\prime }\right)}^{2}$$

(84) $$\Pi_{SC_{i}}^{c\prime }=\frac{1}{b}{\left(q_{i}^{c\prime }\right)}^{2}$$

(85) $$\Pi_{PA_{j}}^{c\prime }=\frac{1-\tau }{b}{\left(q_{j}^{c\prime }\right)}^{2}$$

(86) $$\Pi_{MS_{j}}^{c\prime }=\frac{\tau }{b}{\left(q_{j}^{c\prime }\right)}^{2}$$

(87) $$\Pi_{SC_{j}}^{c\prime }=\frac{1}{b}{\left(q_{j}^{c\prime }\right)}^{2}$$

(88) $$SW_{c}^{\prime }=\frac{a}{\bar{x}}\left(q_{i}^{c\prime }+q_{j}^{c\prime }\right)-\frac{b}{2X}{\left(q_{i}^{c\prime }+q_{j}^{c\prime }\right)}^{2}+\frac{1}{x}q_{i}^{c\prime }q_{j}^{c\prime }-\left[\left(c_{i}+c_{0i}\right)q_{i}^{c\prime }+\left(c_{j}+c_{0j}\right)q_{j}^{c\prime }\right]$$

(89) $$CS_{c}^{\prime }=\frac{a}{\bar{x}}\left(q_{i}^{c\prime }+q_{j}^{c\prime }\right)-\frac{b}{2X}{\left(q_{i}^{c\prime }+q_{j}^{c\prime }\right)}^{2}+\frac{1}{x}q_{i}^{c\prime }q_{j}^{c\prime }-\left(p_{i}^{c\prime }q_{i}^{c\prime }+p_{j}^{c\prime }q_{j}^{c\prime }\right)$$

### 5.3. Hybrid Modeling Scenario: Dual Competing PV Supply Chain Equilibrium and Cooperation without the Government’s Subsidy 

In the hybrid modeling scenario, the PV system assembler *PA_i_* and the module supplier *MS_i_* play the Stackelberg game, *MS_i_* acts as a Stackelberg leader and *PA_i_* acts as a Stackelberg follower; *PA_j_* and *MS_j_* cooperate with each other via Nash bargaining mechanism. *MS_i_* first decides the wholesale price of a module, *PA_i_* then decides the retail price of a PV system to maximize his profit; *MS_j_* and *PA_j_* bargain over the wholesale price of a module to achieve cooperation within the PV supply chain *j*. The PV supply chain *j* then decides the retail price of a PV system to maximize its profit. Finally, the retail price competition occurs in the PV market. The Stackelberg game-Nash bargaining model for the dual competing PV supply chains can be formulated as:

(90) $$\begin{array}{c} \left\{ \begin{array}{c} \underset{w_{j}}{\max}\;{\left[\Pi_{MS_{j}}^{c}\left(w_{j}\right)\right]}^{\tau }{\left[\Pi_{PA_{j}}^{c}\left(w_{j}\right)\right]}^{1-\tau }\\ s.t.\;\Pi_{MS_{j}}^{c}\left(w_{j}\right)+\Pi_{PA_{j}}^{c}\left(w_{j}\right)=\Pi_{SC_{j}}^{c}\left(s\right) \end{array}\right.\\ p_{i}^{d}\left(s\right),p_{j}^{c}\left(s\right),w_{i}^{d}\left(s\right)\;and\;\Pi_{SC_{j}}^{c}\left(s\right)\;are\;derived\;from\;solving\;the\;following\;problem\\ \left\{ \begin{array}{c} \underset{w_{i}}{\max}\;\Pi_{MS_{i}}\left(w_{i}\right)\\ s.t.\;\left\{ \begin{array}{c} \max_{p_{i}}\;\Pi_{PA_{i}}\left(p_{i}\right)\\ \max_{p_{j}}\;\Pi_{SC_{j}}\left(p_{j}\right) \end{array}\right. \end{array}\right. \end{array}$$

Solving this two-stage Stackelberg game-Nash bargaining problem, we can obtain the equilibrium (or bargaining) wholesale price $w_{i}^{d\prime }$ and $w_{j}^{c\prime }$, the equilibrium (or optimal) retail price $p_{i}^{d\prime }$ and $p_{j}^{c\prime }$, and the equilibrium (or optimal) order quantity $q_{i}^{d\prime }$ and $q_{j}^{c\prime }$ as follows:

(91) $$w_{i}^{d\prime }=\frac{ya}{2Z}-\frac{Z\left(c_{0i}+c_{i}\right)-V\left(c_{0j}+c_{j}\right)}{2Z}+c_{i}$$

(92) $$w_{j}^{c\prime }=\tau \left[\frac{\left(2Z+V\right)a}{2\bar{y}Z}-\frac{\left(2Z^{2}-V^{2}\right)\left(c_{j}+c_{0j}\right)-ZV\left(c_{0i}+c_{i}\right)}{2YZ}\right]+c_{j}$$

(93) $$p_{i}^{d\prime }=\frac{\left(Z+W\right)a}{\bar{y}Z}+\frac{WZ\left(c_{0i}+c_{i}\right)+\left(Z+W\right)V\left(c_{0j}+c_{j}\right)}{YZ}$$

(94) $$p_{j}^{c\prime }=\frac{\left(2Z+V\right)a}{2\bar{y}Z}+\frac{\left(Y+2Z\right)W\left(c_{0j}+c_{j}\right)+ZV\left(c_{0i}+c_{i}\right)}{2YZ}$$

(95) $$q_{i}^{d\prime }=\frac{ba}{2\bar{y}}-\frac{b\left[Z\left(c_{0i}+c_{i}\right)-V\left(c_{0j}+c_{j}\right)\right]}{2Y}$$

(96) $$q_{j}^{c\prime }=\frac{b\left(2Z+V\right)a}{2\bar{y}Z}-\frac{b\left[\left(2Z^{2}-V^{2}\right)\left(c_{0j}+c_{j}\right)-ZV\left(c_{0i}+c_{i}\right)\right]}{2YZ}$$

We can also get the optimal (or bargaining) profits of *PA_i_*, *MS_i_*, *SC_i_*, *PA_j_*, *MS_j_*, *SC_j_*, *SW* and *CS* as follows:

(97) $$\Pi_{PA_{i}}^{d\prime }=\frac{1}{b}{\left(q_{i}^{d\prime }\right)}^{2}$$

(98) $$\Pi_{MS_{i}}^{d\prime }=\frac{Y}{bZ}{\left(q_{i}^{d\prime }\right)}^{2}$$

(99) $$\Pi_{SC_{i}}^{d\prime }=\frac{Y+Z}{bZ}{\left(q_{i}^{d\prime }\right)}^{2}$$

(100) $$\Pi_{PA_{j}}^{c}=\frac{1-\tau }{b}{\left(q_{j}^{c\prime }\right)}^{2}$$

(101) $$\Pi_{MS_{j}}^{c\prime }=\frac{\tau }{b}{\left(q_{j}^{c\prime }\right)}^{2}$$

(102) $$\Pi_{SC_{j}}^{c\prime }=\frac{1}{b}{\left(q_{j}^{c\prime }\right)}^{2}$$

(103) $$SW_{cd}^{\prime }=\frac{a}{\bar{x}}\left(q_{i}^{d\prime }+q_{j}^{c\prime }\right)-\frac{b}{2X}{\left(q_{i}^{d\prime }+q_{j}^{c\prime }\right)}^{2}+\frac{1}{x}q_{i}^{d\prime }q_{j}^{c\prime }-\left[\left(c_{i}+c_{0i}\right)q_{i}^{d\prime }+\left(c_{j}+c_{0j}\right)q_{j}^{c\prime }\right]$$

(104) $$CS_{cd}^{\prime }=\frac{a}{\bar{x}}\left(q_{i}^{d\prime }+q_{j}^{c\prime }\right)-\frac{b}{2X}{\left(q_{i}^{d\prime }+q_{j}^{c\prime }\right)}^{2}+\frac{1}{x}q_{i}^{d\prime }q_{j}^{c\prime }-\left(p_{i}^{d\prime }q_{i}^{d\prime }+p_{j}^{c\prime }q_{j}^{c\prime }\right)$$

## 6. Numerical and Sensitivity Analysis

Due to the high conversion efficiency and its popular application, a PV system with modules of 6 × 12 (72) solar cells (max output-power: 335 Watt) for general solutions is used for the numerical analysis of the PV supply chain models developed in Section 4 and Section 5. Using the statistics of the PV industry [32], we can calculate the cost parameters for a PV supply chain in Year 2017: the c-Pi module cost is 194.30 USD/unit, the balance-of-system (BoS) cost is 291.45 USD/unit. The parameters of the demand function are assumed as: *a* = 5000, *b* = 3, *d* = 1. The bargaining power of a MS is set as: *τ* = 0.4 lower than that of a PA. In Table 1, the unit costs for *MS*_1_, *MS*_2_ and *PA*_1_, *PA*_2_ in the dual PV supply chains are set according to the average costs of the PV industry. The two supply chains have a similar cost structure where the supply chain *i* has a one dollar advantage over the supply chain *j* for both module unit costs and BoS unit costs and the same seller-buyer relationship and market size to begin with. The sensitivity analysis is conducted by adding a dollar to the unit costs twice on the supply chain *j* as shown in Table 1: *Sensitivity One* and *Sensitivity Two*.

The numerical and sensitive analysis results of the dual competing PV supply chain equilibrium and cooperation under the social welfare maximization and the results with and without the government’s subsidy are shown and compared in Table 2 and Table 3. The findings from the numerical and sensitivity analysis results are summarized below:

(1) Among the three modeling scenarios, for both PV supply chain *i* and *j*, the wholesale price of a module for the centralized scenario is less than that of the hybrid scenario; and the wholesale price of a module for the hybrid scenario is less than that of the decentralized decision (see Table 2).
(2) Among the three modeling scenarios, for the lower cost PV supply chain *i*, the retail price of its PV system under the centralized scenario is less than that under the decentralized scenario, and the retail price of a PV system under the decentralized scenario is less than that under the hybrid scenario; for the higher cost PV supply chain *j*, the retail price of a PV system under the centralized scenario is more than that under the decentralized scenario, and the retail price of a PV system under the decentralized scenario is more than that under the hybrid scenario (see Table 2).
(3) Among the three modeling scenarios, for the lower cost PV supply chain *i*, the ordering quantity of a PV system under the centralized scenario is larger than that under the decentralized scenario, and the ordering quantity of a PV system under the decentralized scenario is larger than that under the hybrid scenario; for the higher cost PV supply chain *j*, the ordering quantity of a PV system under the centralized scenario is less than that under the decentralized scenario, and the ordering quantity of a PV system under the decentralized scenario is less than that under the hybrid scenario (see Table 2).
(4) Among the three modeling scenarios, for both PV supply chain *i* and *j*, the equilibrium (or optimal) profits of the PV supply chains and their members under the centralized scenario are less than those under the hybrid scenario, and the equilibrium (or optimal) profits of the PV supply chains and their members under the hybrid scenario are less than those under the decentralized scenario (see Table 2).
(5) Among the three modeling scenarios, the equilibrium social welfare under the centralized scenario is more than that under the hybrid scenario, and the equilibrium social welfare under the hybrid scenario is more than that under the decentralized scenario (see Table 2).
(6) Among the three modeling scenarios, the customer’s surplus under the centralized scenario is more than that under the hybrid scenario, and the customer’s surplus under the hybrid scenario is more than that under the decentralized scenario (see Table 2).
(7) Among the three modeling scenarios, the government’s subsidy under the centralized scenario is less than that under the hybrid scenario, and the government’s subsidy under the hybrid scenario is less than that under the decentralized scenario (see Table 2).
(8) When the unit module cost and the unit assembly cost of PV supply chain *i* are fixed, when the unit module cost and the unit assembly cost of PV supply chain *j* increases by a dollar, the equilibrium (or optimal) profits of PV supply chain *i* and *j* and their members will decrease, the trend also holds for the equilibrium social welfare, the customer’s surplus and the government’s subsidy (see Table 2). Nevertheless, the decreases of these values are trivial, i.e., the impact on the profits due to a small increase of the unit costs is very limited. This shows that when the cost structures of PV supply chains are very close to each other, the pricing decisions of these supply chains in the market will also be very close.
(9) Among the three modeling scenarios, the equilibrium (or optimal) profits of PV supply chain *i* and *j* and their members under the government’s subsidy are more than those without the subsidy. The trend holds also for the equilibrium social welfare and the customer’s surplus (see Table 2 and Table 3).
(10) Between the subsidy and no subsidy, the dual PV supply chains have the largest incentive to seek subsidies and pursue the decentralized strategy to achieve their maximum returns; however, the government would need to pay the maximal subsidy budget. The best option for the government would be to encourage the dual PV supply chains to adopt a centralized strategy since this will not only maximize the social welfare but also, at the same time, minimize the public subsidy. If there is no public subsidy, the optimal strategy for the dual supply chains would be to pursue the centralized strategy to gain the most returns for the supply chain members and the supply chains comparing to the decentralized or the hybrid strategies. The centralized strategy also creates the maximal social welfare when there is no subsidy.

## 7. Managerial and Policy Implications

Table 4 and Table 5 consolidate the pricing, total order quantity, profits, social welfare and total subsidy statistics of Table 2 and Table 3 to compare their differences due to the modeling scenarios (or supply chain strategies) to facilitate the derivation of the managerial and policy insights and several policy recommendations.

### 7.1. No Subsidy

It is obvious that taking the centralized strategy for both supply chain *i* and *j* can create the highest total PV system demands, total supply chain profits and social welfare in the dual supply chain system when there is no government subsidy (see Table 5). Under the Bertrand competition, two supply chains compete by pricing the PV systems at the same level to equally share the market demands in the centralized dual supply chains (see Table 4). However, *MSi* and *MSj* are not given good enough the wholesale prices in the bargaining process due to the lower bargaining power (0.4) as the suppliers. If both module suppliers have the market power powers within their own supply chains, they would have higher motivations to take the decentralized strategy to charge much higher wholesale prices and maximize their own profits rather than to be a “nice guy” to sacrifice for the goodness of the supply chains and the society. If one supplier has the market power and the other does not, the hybrid strategy would become the best supply chain strategy for the dual supply chains but the total supply chain profits and the social welfare would be in the middle of those created by the other two supply chain strategies.

Since the competition now is between two supply chains, the supply chain members in each supply chain should take the cooperative approach to create the highest demands in the market and maximize the total supply chain profits. Thus, under the case without the public subsidy, it is the best from the social welfare maximization and the total supply chain perspective for the dual supply chains to adopt a centralized strategy.

Therefore, an innovative mechanism should be developed in the dual supply chains to provide more incentives to the module suppliers, such as offering higher wholesale prices, revenue-sharing by the PV assemblers, to ensure the supply chains would move towards a centralized supply chain system. In other words, new methodologies or innovations should be developed to deal with the problem that the Nash bargaining under the Bertrand competition could not resolve in this study. This provides a new direction for the future study of the dual supply chain competition.

### 7.2. Subsidy

When the public subsidy is available, the centralized and the decentralized strategies create the same amount of the social welfare value which is better than that of the hybrid strategy. However, the decentralized strategy consumes three times more subsidy money than that of the centralized strategy, in the mean times, also takes away three times more supply chain profits than that of the centralized strategy.

Since the dual supply chains, with the subsidy money, can sell PV systems to the market with prices much lower than their costs, much more demand can be created. This serves well the purpose of promoting the solar energy industry. However, the decentralized dual supply chains reap too much subsidy money due to the market power of the module suppliers. Thus, from the public policy perspective, gearing the dual supply chains to take a centralized strategy would serve the public interest best since this not only creates the largest social welfare effect but also uses the least amount of subsidy money (comparing to the other two supply chain strategies).

To avoid over-subsidizing to the PV industry, it would be wise for a government to set a threshold or ceiling to the subsidy based on the centralized modeling results and develop policies that can encourage the members in PV supply chains to cooperate and form centralized supply chains.

An effective subsidy mechanism must integrate the market users and the PV supply chains. There are several directions to design an effective subsidy mechanism [33]. Shared solar energy arrangements, for example, allow several energy customers to share the benefits of one local solar energy power plant. This is sometimes called community solar. The community solar segment is on the cusp of becoming a mainstream driver of U.S. solar market growth. Starting in 2017, community solar is expected to consistently drive 20–25% of the annual non-residential PV market and become a half-gigawatt annual market by 2019 [34]. The shared solar project pools investments from multiple members of a community and provides power and/or financial benefits in return. A centralized PV supply chain can supply the PV systems to the local solar energy power plant who can utilize the subsidy incentives to install the PV systems.

Whenever possible, incentives should be awarded to the high-performing PV system assemblers and their supply chains. Direct incentives can be adjusted to encourage the PV system installations that maximize peak energy production. A performance-based incentive (PBI) is based on the actual energy production of a PV system. PBIs inherently incentivize optimal system design and encourage active, ongoing maintenance [35,36]. PBIs can not only encourage the PV system installers to cooperate with the high-performing PV supply chains but also discourage installers to put up shoddy systems provided by the low-performing PV supply chains as quickly as possible to capture the incentives. This can then build up a healthier and growing solar energy market.

## 8. Conclusions

This study has explored the dual PV supply chain competition under the Bertrand competition assumption by three game-theoretical modeling scenarios (or supply chain strategies) considering either the public subsidy or no subsidy from a social welfare maximization perspective. A numerical analysis complemented by two sensitivity analyses provides a better understanding of the pricing and quantity decision dynamics in the dual supply chains under three different supply chain strategies and the corresponding outcomes regarding the total supply chain profits, the social welfare and the required total subsidies.

The key findings reveal that if there is no public subsidy, the optimal strategy for the dual supply chains would be to pursue the centralized strategy to gain the most returns for the supply chain members and the supply chains comparing to the decentralized or the hybrid strategies. The centralized strategy also creates the maximal social welfare when there is no subsidy. Furthermore, if there are public subsidies, the dual PV supply chains have the strongest intention to pursue the decentralized strategy to achieve their maximal returns rather than the centralized strategy that would achieve the maximal social welfare; however, the government would need to pay for the maximal subsidy budget. Thus, the best option for the government would be to encourage the dual PV supply chains to adopt a centralized strategy since this will not only maximize the social welfare but also, at the same time, minimize the public subsidy.

To prevent over-subsidizing to the PV industry, this study recommends a government could set a threshold or ceiling to the subsidy based on the centralized modeling results and develop policies that can encourage the members in the PV supply chains to cooperate and form centralized supply chains. Several subsidy policies such as shared solar energy arrangements and performance-based incentives (PBIs) are proposed to integrate the market users and the PV supply chains. With a smart subsidy policy, the PV industry can make the best use of the subsidy budget and grow in a sustainable way to support the highly demanded solar power generation in many countries trying very hard to increase the proportion of their clean energy to combat the global warming effect.

In conclusion, this study serves as a pioneering study into the dual PV supply chain research which is very limited in the PV management and policy study literature. The findings and several untended issues, such as how to set and allocate the subsidy threshold, provide a foundation for the future PV supply chain studies. With the methodology developed in this study looking at the dual PV supply chains, it is possible to extend the PV supply chain study to look at more than two competing supply chains which present an even more realistic picture in the PV industry and also a more challenging research problem for the future.

## Figures and Tables

**Table 1:** Cost and demand parameters in the dual PV supply chains and for the sensitivity analysis.

Parameters		*SC_i_*	*SC_j_*
*c_i_*	c-Pi module cost (USD/unit)	194.30	195.30
*c*_0*i*_	Assembling (BoS) cost (USD/unit)	291.45	292.45
*a*	Maximum potential market size for PV supply chain *i*	5000	5000
*b*	Reaction extent of the demand w.r.t the change of the retail price	3	3
*d*	Reaction degree of the demand w.r.t the change of the competitor’s retail price	1	1
*τ*	Bargaining power	0.4	0.4
*c_i_*	*Sensitivity One*	194.30	19630
*c*_0*i*_		291.45	293.45
*c_i_*	*Sensitivity Two*	194.30	197.30
*c*_0*i*_		291.45	294.45

**Table 2:** Numerical and sensitive analysis results of dual competing PV supply chains under social welfare maximization.

Analysis	Original			*Sensitivity One*			*Sensitivity Two*		
Unit Cost	(*c_i_* = 194.3, *c*_0*i*_ = 291.45; *c_j_* = 195.30, *c*_0*j*_ = 292.45)			(*c_i_* = 194.3, *c*_0*i*_ = 291.45; *c_j_* = 196.30, *c*_0*j*_ = 293.45)			(*c_i_* = 194.3, *c*_0*i*_ = 291.45; *c_j_* = 197.30, *c*_0*j*_ = 294.45)		
Scenario	Decentralized	Centralized	Hybrid	Decentralized	Centralized	Hybrid	Decentralized	Centralized	Hybrid
Solutions									
$s_{*}$	4105.45	1342.17	1940.73	4103.41	1341.50	1939.60	4101.37	1340.83	1938.48
$w_{i}^{*}$	2958.12	731.40	1823.00	2957.29	731.36	1822.71	2956.46	731.32	1822.42
$p_{i}^{*}$	486.55	486.32	964.80	487.36	486.89	965.50	488.16	487.46	966.20
$q_{i}^{*}$	4027.29	4028.21	2373.24	4026.08	4027.93	2372.82	4024.86	4027.64	2372.41
$w_{j}^{*}$	2958.04	731.94	883.55	2957.13	732.44	883.97	2956.22	732.95	884.39
$p_{j}^{*}$	486.95	487.18	267.65	488.14	488.61	269.32	489.34	490.04	271.00
$q_{j}^{*}$	4025.71	4024.79	5161.86	4022.92	4021.07	5157.53	4020.14	4017.36	5153.19
Supply Chain Profits and Social Welfare									
$\Pi_{PA_{i}}^{*}$	5,406,349	3,245,302	1,877,427	5,403,094	3,244,842	1,876,766	5,399,841	3,244,381	1,876,105
$\Pi_{MS_{i}}^{*}$	11,130,718	2,163,535	3,865,291	11,124,017	2,163,228	3,863,930	11,117,319	2,162,921	3,862,570
$\Pi_{SC_{i}}^{*}$	16,537,066	5,408,837	5,742,718	16,527,111	5,408,070	5,740,696	16,517,159	5,407,302	5,738,675
$\Pi_{PA_{j}}^{*}$	5,402,120	3,239,780	5,328,969	5,394,641	3,233,803	5,320,016	5,387,167	3,227,832	5,311,071
$\Pi_{MS_{j}}^{*}$	11,122,012	2,159,853	3,552,646	11,106,614	2,155,869	3,546,677	11,091,227	2,151,888	3,540,714
$\Pi_{SC_{j}}^{*}$	16,524,132	5,399,633	8,881,615	16,501,255	5,389,672	8,866,694	16,478,394	5,379,719	8,851,785
$SW_{*}$	8,106,353	8,106,354	7,584,010	8,098,306	8,098,311	7,574,446	8,090,266	8,090,277	7,564,892
$CS_{*}$	8,106,351	8,106,352	7,583,256	8,098,301	8,098,303	7,572,935	8,090,255	8,090,260	7,562,621
$TS_{*}$	33,061,197	10,808,468	14,623,578	33,028,361	10,797,734	14,605,878	32,995,542	10,787,004	14,588,189

**Table 3:** Numerical and sensitive analysis results of dual competing PV supply chains without the government’s subsidy.

Analysis	Original			*Sensitivity One*			*Sensitivity Two*		
Unit Cost	(*c_i_* = 194.3, *c*_0*i*_ = 291.45; *c_j_* = 195.30, *c*_0*j*_ = 292.45)			(*c_i_* = 194.3, *c*_0*i*_ = 291.45; *c_j_* = 196.30, *c*_0*j*_ = 293.45)			(*c_i_* = 194.3, *c*_0*i*_ = 291.45; *c_j_* = 197.30, *c*_0*j*_ = 294.45)		
Scenario	Decentralized	Centralized	Hybrid	Decentralized	Centralized	Hybrid	Decentralized	Centralized	Hybrid
Solutions									
$w_{i}^{*}$	1104.05	516.65	1023.87	1104.14	516.72	1024.05	1104.23	516.79	1024.23
$p_{i}^{*}$	1837.38	1291.62	1718.26	1837.51	1291.79	1718.52	1837.64	1291.96	1718.78
$q_{i}^{*}$	1325.64	2417.61	1208.81	1325.77	2418.13	1209.06	1325.90	2418.64	1209.32
$w_{j}^{*}$	1103.97	517.19	545.63	1103.98	517.8	546.25	1103.98	518.41	546.87
$p_{j}^{*}$	1837.77	1292.48	1363.58	1838.30	1293.51	1364.63	1838.82	1294.54	1365.67
$q_{j}^{*}$	1324.06	2414.19	2627.50	1322.62	2411.27	2624.64	1321.17	2408.36	2621.77
Supply Chain Profits and Social Welfare									
$\Pi_{PA_{i}}^{*}$	585,770	1,168,971.77	487,072	585,885	1,169,469.16	487,279	585,999	1,169,966.65	487,486
$\Pi_{MS_{i}}^{*}$	1,205,998	779,314.51	1,002,794	1,206,233	779,646.11	1,003,221	1,206,469	779,977.77	1,003,648
$\Pi_{SC_{i}}^{*}$	1,791,768	1,948,286.28	1,489,866	1,792,118	1,949,115.26	1,490,500	1,792,469	1,949,944.42	1,491,134
$\Pi_{PA_{j}}^{*}$	584,379	1,165,658.53	1,380,756	583,104	1,162,845.98	1,377,743	581,829	1,160,036.83	1,374,732
$\Pi_{MS_{j}}^{*}$	1,203,133	777,105.69	920,504	1,200,507	775,230.65	918,495	1,197,884	773,357.88	916,488
$\Pi_{SC_{j}}^{*}$	1,787,512	1,942,764.22	2,301,260	1,783,611	1,938,076.63	2,296,238	1,779,714	1,933,394.71	2,291,220
$SW_{*}$	4,456,892	6,809,337.64	5,756,581	4,452,470	6,802,582.88	5,749,135	4,448,052	6,795,836.87	5,741,697
$CS_{*}$	877,612	2,918,287.14	1,965,455	876,741	2,915,390.98	1,962,397	875,870	2,912,497.74	1,959,342

**Table 4:** Pricing of the supply chains under different modeling scenarios: subsidy vs. no subsidy.

Subsidy	Modeling Scenario	*SC_i_*			*SC_j_*			Total SC Order Quantity	Total SC Profits	Social Welfare	Total Subsidy
		*W_i_*	*P_i_*	*Q_i_*	*W_j_*	*P_j_*	*Q_j_*				
Subsidy	Centralized	731.40	486.32	4028.21	731.94	487.18	4024.79	8053.00	10,808,470	8,106,354	10,808,468
	Decentralized	2958.12	486.55	4027.29	2958.04	486.95	4025.71	8053.00	33,061,198	8,106,353	33,061,197
	Hybrid	1823.00	964.80	2373.24	883.55	267.65	5161.86	7535.10	14,624,332	7,584,010	14,623,578
No subsidy	Centralized	516.65	1291.62	2417.61	517.19	1292.48	2414.19	4831.80	3,891,052	6,809,338	NA
	Decentralized	1104.05	1837.38	1325.64	1103.97	1837.77	1324.06	2649.70	3,579,280	4,456,892	NA
	Hybrid	1023.87	1718.26	1208.81	545.63	1363.58	2627.50	3836.31	3,791,126	5,756,581	NA

**Table 5:** Returns to the supply chains and society under different modeling scenarios: subsidy vs. no subsidy.

Subsidy	Modeling Scenario	*SC_i_*			*SC_j_*			Total SC Order Quantity	Total SC Profits	Social Welfare	Total Subsidy
		MS Profits	PA Profits	SC Profits	MS Profits	PA Profits	SC Profits				
Subsidy	Centralized	2,163,535	3,245,302	5,408,837	2,159,853	3,239,780	5,399,633	8053.00	10,808,470	8,106,354	10,808,468
	Decentralized	11,130,718	5,406,349	16,537,066	11,122,012	5,402,120	16,524,132	8053.00	33,061,198	8,106,353	33,061,197
	Hybrid	3,865,291	1,877,427	5,742,718	3,552,646	5,328,969	8,881,615	7535.10	14,624,332	7,584,010	14,623,578
No subsidy	Centralized	779,315	1,168,972	1,948,287	777,106	1,165,659	1,942,765	4831.80	3,891,052	6,809,338	NA
	Decentralized	1,205,998	585,770	1,791,768	1,203,133	584,379	1,787,512	2649.70	3,579,280	4,456,892	NA
	Hybrid	1,002,794	487,072	1,489,866	920,504	1,380,756	2,301,260	3,8,36.31	3,791,126	5,756,581	NA
